# m^6^A RNA Modification Controls HTLV‐1 Tax and Host Gene Expression

**DOI:** 10.1111/gtc.70054

**Published:** 2025-10-07

**Authors:** Rei Gibu, Kodai Gibu, Kako Suzuki, Yuetsu Tanaka, Kaoru Uchimaru, Makoto Yamagishi

**Affiliations:** ^1^ Laboratory of Viral Oncology and Genomics, Department of Computational Biology and Medical Sciences, Graduate School of Frontier Sciences The University of Tokyo Tokyo Japan; ^2^ Geological Survey of Japan National Institute of Advanced Industrial Science and Technology (AIST) Ibaraki Japan; ^3^ Department of Ecosystem Studies, Graduate School of Agricultural and Life Sciences The University of Tokyo Tokyo Japan; ^4^ Laboratory of Hematoimmunology, Graduate School of Health Sciences University of the Ryukyus Okinawa Japan; ^5^ Laboratory of Tumor Cell Biology, Department of Computational Biology and Medical Sciences, Graduate School of Frontier Sciences The University of Tokyo Tokyo Japan

## Abstract

Human T‐cell Leukemia Virus Type 1 (HTLV‐1) is a pathogenic human retrovirus that is responsible for intractable diseases such as adult T‐cell leukemia–lymphoma (ATL), a malignancy with a poor patient prognosis. Although recent studies have delineated several genomic, epigenomic, and transcriptomic abnormalities associated with HTLV‐1, to date the importance of epitranscriptomic modifications, particularly *N* ^*6*^‐methyladenosine (m^6^A), remains unclear. Here, we showed that the HTLV‐1 RNA genome undergoes m^6^A modification, thereby suggesting that these modifications act as bidirectional regulators of both viral and host processes. Moreover, targeted depletion of m^6^A modification within the viral transactivator HTLV‐1 Tax resulted in markedly destabilized Tax mRNA, attenuated Tax protein abundance, and suppression of downstream expression of host genes including *IL2RA* and *TXN*. Overall, these findings suggest that m^6^A methylation is an essential determinant of the HTLV‐1 life cycle, and understanding it may offer mechanistic insight into viral latency and present novel avenues for therapeutic intervention and prophylaxis.

## Graphical abstract

Our study shows that m^6^A regulation is critical for controlling HTLV‐1, shaping both viral transcripts and host gene programs, thereby illuminating latency mechanisms and suggesting tractable targets for therapy and prevention.

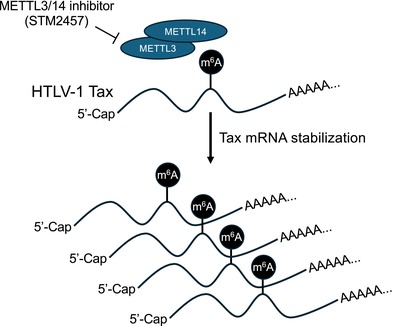

## 1 Introduction

Human T‐cell Leukemia Virus Type‐1 (HTLV‐1) is a retrovirus that infects CD4‐positive T cells. HTLV‐1 enters a latent phase post‐infection, and approximately 5% of infected individuals eventually develop conditions such as adult T‐cell leukemia–lymphoma (ATL), HTLV‐1‐associated myelopathy (HAM), or HTLV‐1‐associated uveitis (HU). Relative to other forms of leukemia, ATL is a particularly severe form of hematological malignancy. Moreover, numerous studies have reported specific genomic, epigenomic, and transcriptomic aberrations that are associated with HTLV‐1 (Kataoka et al. 2015; Kogure et al. 2022; Yamagishi et al. 2021; Mizuike et al. 2025; Kobayashi et al. 2014), but to date a detailed understanding of its pathogenic mechanisms remains unclear.

HTLV‐1 expresses two functional genes, *tax* and *hbz*, both of which have been extensively studied. The Tax protein acts as a transcriptional activator of other HTLV‐1 genes (Seiki et al. 1985) and is implicated in tumorigenesis and epigenetic alterations (Hasegawa et al. 2006; Ohsugi et al. 2007; Fujikawa et al. 2016). Tax is highly expressed during the early stages of infection but later is suppressed via epigenetic silencing or the emergence of clones containing deletions in the Tax coding region (Taniguchi et al. 2005; Tamiya et al. 1996), thereby leading to clonal proliferation. In contrast, HBZ is expressed during all infection phases and can induce the expression of FoxP3, a master transcription factor for regulatory T cells (Tregs) (Zhao et al. 2011). Furthermore, *hbz*‐transgenic mice may develop cancer (Satou et al. 2011).

The regulation of major viral factors capable of influencing both viral replication and host‐cell physiology has been characterized mainly on the transcriptional level and has included novel studies of promoter (e.g., long‐terminal repeats, LTRs) (Fujisawa et al. 1985) and enhancer regions (Matsuo et al. 2022). In contrast, much less is known regarding post‐transcriptional control of viral transcripts, which represents a critical unresolved question for elucidating mechanisms governing viral gene expression.

*N* ^6^‐methyladenosine (m^6^A) modification of RNA is characterized by the consensus motif DRACH (D = A/G/U, *R* = A/G, H = A/C/U). Moreover, it is a reversible process mediated by m^6^A writers, such as the METTL3 complex, and erasers such as FTO or ALKBH5. In general, m^6^A reader proteins determine the fate of m^6^A‐modified RNA by influencing its decay or translation, two key forms of post‐transcriptional regulation (Dominissini et al. 2012; Jia et al. 2011; Zheng et al. 2013; Meyer and Jaffrey 2017; Duan et al. 2024).

Post‐transcriptional regulation via m^6^A modification is a crucial factor in both cancer and in viral life cycles (McFadden and Horner 2021). For example, the human retrovirus HIV‐1 undergoes m^6^A modification, which affects nuclear export, virion formation, and latent infection (Lichinchi et al. 2016; Tsai et al. 2021; Mishra et al. 2024). Moreover, in various cancers, abnormal expression of writers, erasers, and readers for m^6^A modifications has been linked to a variety of features, including tumor invasion, metastasis, and tumor growth (He et al. 2019). Recent studies have reported that HTLV‐1 RNA is modified by m^6^A, and that some m^6^A reader proteins are involved in HTLV‐1 RNA regulation (King et al. 2025). However, to date, it remains unclear how m^6^A‐modified HTLV‐1 RNA affects the control of host genes.

In this study, we investigated m^6^A modification of the HTLV‐1 genome in HTLV‐1‐infected cell lines and conducted further exploration of its functional implications.

## 2 Results

### 2.1 HTLV‐1 RNA Contains m^6^A Modification

**FIGURE 1:**
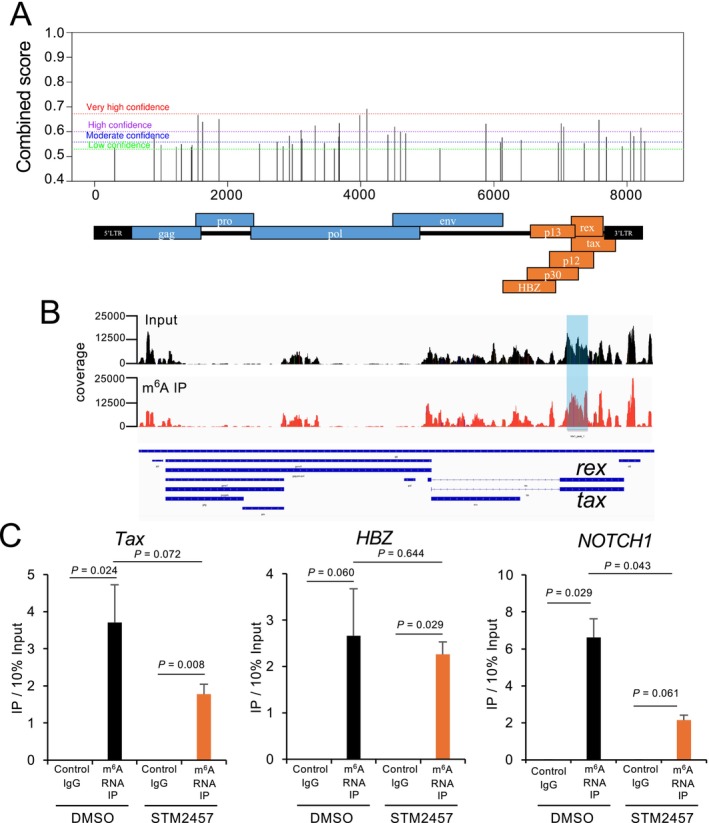
Prediction and validation of m^6^A modification sites in HTLV‐1 RNA. (A) The results of predicting the m^6^A modification sites in the HTLV‐1 genome. (B) m^6^A modifications of HTLV‐1 RNA using public MeRIP‐seq data for the HTLV‐1 infected cell line MT‐4. Read‐depth tracks are displayed above; the HTLV‐1 genomic organization is depicted below. (C) The results of RIP‐qPCR using the HTLV‐1‐infected cell line C91/PL. As a negative control, m^6^A levels were inhibited with the m^6^A writer inhibitor STM2457. *n* = 3, mean ± SD.

First, we predicted potential m^6^A modification sites in the HTLV‐1 genome (NCBI Reference Sequence: NC_001436.1) using the sequence‐based prediction tool SRAMP (Zhou et al. 2016). In total, 44 m^6^A modification sites were predicted in HTLV‐1 genomic RNA, including in structural genes (e.g., *gag*, *pol*, and *env*), regulatory genes (e.g., *tax* and *rex*), and accessory genes (e.g., *p12*, *p13*, *p30*, and *hbz*). Taken together, these findings suggest the potential for m^6^A modification of both structural and functional gene regions (Figure 1A).

Next, we investigated m^6^A modifications of HTLV‐1 RNA using public MeRIP‐seq data for the HTLV‐1 infected cell line MT‐4 (Lichinchi et al. 2016). We extracted HTLV‐1 genome data and reanalyzed m^6^A modification sites. In doing so, we identified a peak between bases 7109–7439 of the 8507 bp HTLV‐1 genome. This region encodes Tax/Rex, and the MeRIP‐seq data indicated that this coding region contains m^6^A modifications (Figure 1B). Moreover, approximately 1–3 m^6^A modification sites have been detected per host gene in previous studies (Dominissini et al. 2012), a finding that is consistent with our secondary data analysis of HTLV‐1 genes.

Based on these results, we conducted RNA immunoprecipitation followed by quantitative PCR (RIP‐qPCR) analyses on the HTLV‐1 infected cell line C91/PL. For RIP‐qPCR, cells were first pre‐treated with the METTL3/14 inhibitor STM2457 72 h before RIP; this sample was used as a negative control. Next, m^6^A‐modified transcripts were isolated using a mRNA‐specific anti‐m^6^A antibody, and the resulting precipitated HTLV‐1 *tax* and *hbz* mRNAs were quantified. Relative to control IgG samples, DMSO‐treated samples immunoprecipitated with the anti‐m^6^A antibody showed significant enrichment of *tax* mRNA as well as *NOTCH1* mRNA, consistent with m^6^A modification of both viral and host transcripts (Figure 1C). However, this enrichment was reduced by STM2457 pretreatment, supporting the specificity of the assay. Taken together, our in silico predictions and the experimental evidence reported here support the hypothesis that HTLV‐1 *tax* and *hbz* mRNAs contain m^6^A modifications.

### 2.2 Stability of HTLV‐1 Tax Is Downregulated by m^6^A Depletion

**FIGURE 2:**
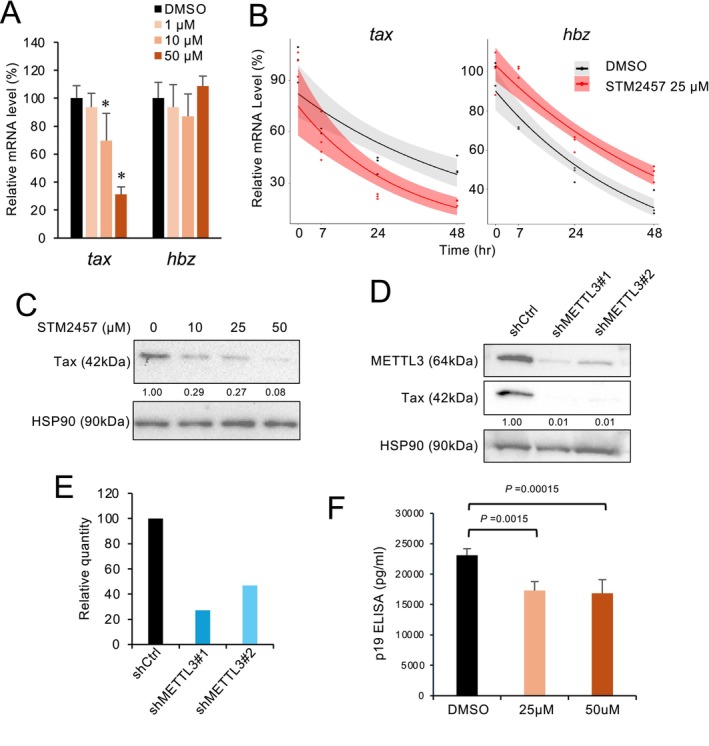
Behavior of HTLV‐1 genes upon treatment with METTL3/14 inhibitor STM2457. (A) C91/PL were treated with the METTL3/14 inhibitor STM2457 for 72 h. Expression levels of HTLV‐1 RNA were evaluated by qRT‐PCR. *n* = 3, mean ± SD, *p < 0.05. (B) HTLV‐1 mRNA stability was analyzed by RNA decay assay using actinomycin D (*n* = 3). Time‐lapse kinetics of HTLV‐1 RNA were shown as linear modeling with a Gamma distribution. (C) C91/PL cells were treated with STM2457 for 72 h. The protein expression of Tax was detected by Western blotting. HSP90 served as a loading control. Quantified expression levels are shown below the panel. (D) Western blot analysis of METTL3 and Tax in METTL3 knockdown C91/PL cells. Quantified expression levels are shown below the panel. (E) *Tax* mRNA levels in METTL3 knockdown cells were measured by qRT‐PCR. (F) C91/PL cells were treated with STM2457, followed by quantification of viral antigen release (p19 gag) by ELISA. *n* = 5, mean ± SD.

Intracellular m^6^A‐modified mRNAs are first recognized by the reader protein YTHDF2, then recruited to the CCR4‐NOT deadenylase complex, thereby promoting RNA decay (Du et al. 2016; Dominissini et al. 2012). To examine the functional impact of m^6^A, C91/PL cells were treated with increasing concentrations of STM2457 to deplete m^6^A, then we quantified the abundance of viral mRNA present in samples. Unexpectedly, Tax mRNA levels decreased in a dose‐dependent manner, while *hbz* mRNA levels remained unchanged. In contrast, although we observed *hbz* mRNA was also m^6^A‐modified, no differential expression was detected (Figure 2A).

To evaluate how m^6^A depletion affects viral RNA stability, we exposed C91/PL cells to STM2457, then halted transcription using actinomycin D. Under these conditions, the stability of Tax mRNA was strongly reduced following STM2457 treatment, whereas the stability of *hbz* mRNA increased (Figure 2B). We also performed immunoblotting in parallel to show that Tax protein levels declined following exposure to STM2457 (Figure 2C).

To corroborate our inhibitor‐based findings, we performed METTL3 knockdown. We generated two independent shRNA‐expressing cell lines and achieved robust METTL3 depletion. In METTL3‐depleted cells, Tax expression was clearly reduced (Figure 2D,E). Concordant changes at the RNA and protein levels support a model in which HTLV‐1 Tax expression is regulated post‐transcriptionally in an m^6^A‐dependent manner.

### 2.3 m^6^A Depletion Impairs Progeny Viral Production

Given that Tax is indispensable for transactivating the HTLV‐1 5′‐LTR promoter, alterations in the abundance of Tax may affect viral production. Next, we attempted to determine whether m^6^A depletion influences progeny virus production (Cann et al. 1985; Fujisawa et al. 1986). To do so, C91/PL cells were treated with STM2457, after which viral antigen release (*p19* gag) was quantified by ELISA. We found that exposure to STM2457 significantly reduced extracellular *p19 gag* levels (Figure 2F), indicating suppressed viral gene expression. Collectively, these data suggest that the m^6^A modification plays a critical role in the HTLV‐1 life cycle by sustaining Tax‐mediated transcriptional activation.

### 2.4 m^6^A Depletion Alters the Expression of Genes Important for HTLV‐1 Infection

HTLV‐1 Tax interacts with many host factors and significantly affects the regulation of host gene expression. It does so mainly by activating the NF‐κB signaling pathway and reprogramming the epigenetic architecture of the genome (Murakami et al. 1995; Mizuike et al. 2025). Therefore, we investigated changes in host gene expression after a 3‐day STM2457 treatment, using RNA sequencing (RNA‐seq).

**FIGURE 3:**
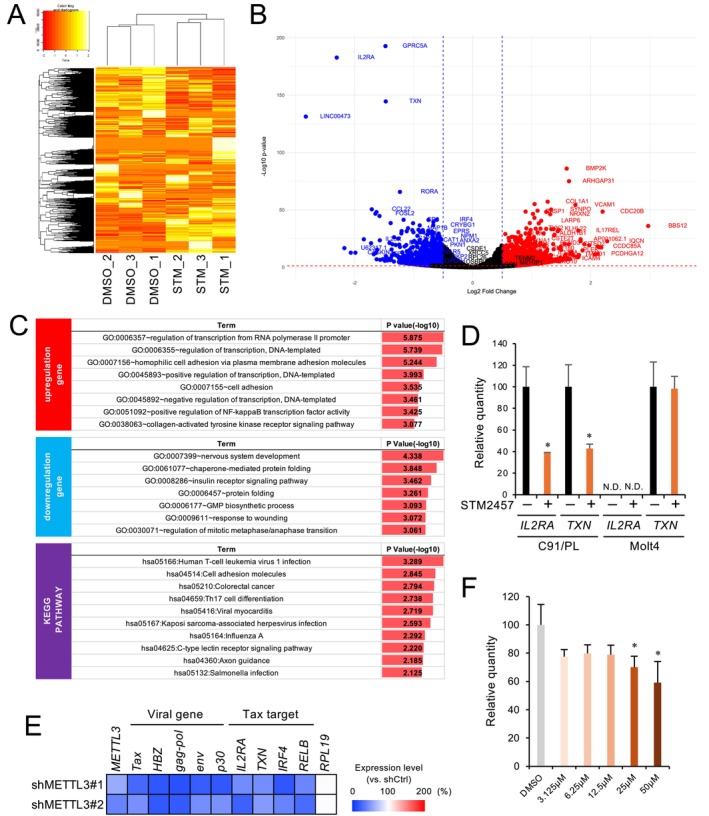
Comprehensive analysis of gene expression upon treatment with METTL3/14 inhibitor STM2457. (A) Heatmap of gene expression pattern after exposure to STM2457. Each column represents the control group treated with DMSO and the test group treated with STM (STM2457). (B) Volcano plot showing comparison between control and inhibitor‐treated groups. Genes with increased expression (Log_2_FC > 0.5, *p*_adj_ < 0.05) are shown in red, while genes with decreased expression (Log_2_FC < −0.5, *p*_adj_ < 0.05) are shown in blue. (C) Results of Gene Ontology and KEGG PATHWAY analyses. Differentially expressed “upregulated genes” (Log_2_FC ≥ 0.5, *p*_adj_ < 0.05) and “downregulated genes” (Log_2_FC ≤ −0.5, *p*_adj_ < 0.05) are analyzed, respectively. KEGG PATHWAY analysis was performed using both upregulated and downregulated genes. (D) C91/PL and Molt4 cells were treated with STM2457; expression levels of Tax target genes were quantified by qRT‐PCR (*n* = 3; mean ± SD, *p < 0.05). N.D., not detected. (E) Relative expression levels of viral genes and Tax targets in METTL3‐knockdown C91/PL cells, as measured by qRT‐PCR, are shown in a heatmap. (F) C91/PL cells were cultured for 3 days with the indicated concentrations of STM2457. Cell viability was assessed by WST‐8 assay (*n* = 3; mean ± SD, *p < 0.05 vs. DMSO).

Hierarchical clustering of results from the global transcriptome reveals that STM2457 treatment markedly reshapes the overall gene expression landscape of HTLV‐1 infected cell lines (Figure 3A). Next, we conducted a Differential Expression Gene (DEG) analysis and identified 2246 differentially expressed genes. These genes are listed in Table S1 and Figure 3B. We also performed Gene Ontology (GO) analysis, which revealed that some upregulated genes were associated with transcriptional regulation and cell adhesion (Figure 3C). Downregulated genes are associated with nervous system development and protein folding.

Next we input all DEGs identified here into a KEGG pathway analysis. The most significantly altered pathway was Human T‐cell Leukemia Virus type 1 infection (Figure 3C and Figure S1). Moreover, *IL2RA* (CD25), a tumor marker of ATL (Kamihira et al. 1994), *TXN* (Thioredoxin), which binds Tax at the gene promoter region (Masutani et al. 1996), and *VCAM1* (Vascular Cell Adhesion Molecule 1), a key driver of syncytium formation (Hildreth et al. 1997), all of which exhibited significant differential expression. The STM2457‐dependent downregulation of *IL2RA* and *TXN* was specifically detected in C91/PL, but not in HTLV‐1‐negative Molt4 cells (Figure 3D). Moreover, METTL3 knockdown confirmed the consistent downregulation of Tax target genes (Figure 3E).

Given that STM2457 suppresses both Tax expression and viral production, we examined its impact on cell growth and observed a dose‐dependent reduction (Figure 3F). Taken together, these results further show that m^6^A deficiency alters the expression of host genes critical to HTLV‐1 infection.

### 2.5 CD25 Expression Is Downregulated by STM2457

**FIGURE 4:**
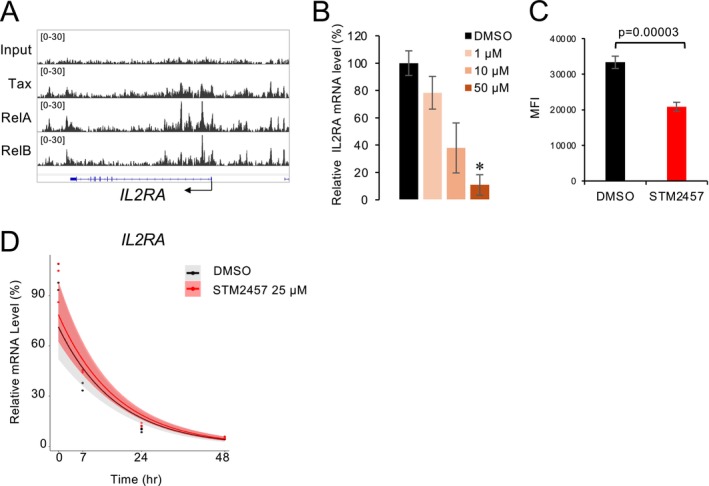
Gene expression changes of HTLV‐1 Tax downstream gene *IL2RA* (CD25) upon treatment with METTL3/14 inhibitor STM2457. (A) IGV tracks of ChIP‐seq signal for input, Tax, RelA, and RelB at the *IL2RA* locus in C91/PL cells. (B) *IL2RA* (CD25) RNA expression level in HTLV‐1‐infected C91/PL cells treated with the METTL3/14 inhibitor STM2457 assessed by qRT‐PCR. *n* = 3, mean ± SD, *p < 0.05. (C) Cell surface expression of CD25 level was analyzed by flow cytometry (*n* = 5, mean ± SD). The *Y*‐axis represents the Mean Fluorescence Intensity (MFI). (D) *IL2RA* mRNA stability was analyzed by RNA decay assay using actinomycin D (*n* = 3).

IL2RA (CD25) is one of the cell surface markers of both HTLV‐1 infected cells and ATL cells, and its expression is dominantly induced by Tax (Ballard et al. 1988). Moreover, Tax is a potent activator of the NF‐κB pathway. Archival ChIP‐seq data (Mizuike et al. 2025) showed binding of Tax and NF‐κB components at the promoter region of *IL2RA* in the Tax‐expressing C91PL cells (Figure 4A). Moreover, exposure of the C91/PL to STM2457, followed by subsequent qRT‐PCR and flow cytometry experiments, showed that lowering Tax expression also reduced *IL2RA* transcript abundance and CD25 surface density (Figure 4B,C). Consistent with the results using the inhibitor, METTL3 knockdown also reduced *IL2RA* expression (Figure 3E). Since *IL2RA* mRNA stability remained unchanged (Figure 4D), we interpret this finding as meaning that such downregulation is attributable to diminished Tax activity rather than as a direct effect of m^6^A depletion.

## 3 Discussion

The impact of m^6^A modification on HTLV‐1 RNA and its effects on the host have become clearer in the recent past. Furthermore, this study suggests that the HTLV‐1 genome does contain m^6^A modification, thereby confirming the findings of another report (Figure 1). Moreover, we have experimentally demonstrated that *tax* mRNA is modified by m^6^A (Figure 1). This finding is consistent with recent reports investigating the presence of m^6^A modifications and RNA binding proteins in HTLV‐1 RNA (King et al. 2025). In general, Tax functions as a transcription factor for HTLV‐1 genes. It binds to the 5′ LTR to initiate transcription of structural genes such as *gag‐pro‐pol* and *env*, as well as functional genes, including *tax* itself and *p30* (Berneman et al. 1992). Overall, our data suggest that m^6^A modification may act to regulate HTLV‐1 gene expression, given its potential impact on the expression of structural genes.

Furthermore, m^6^A modification appears to influence host genes, as evidenced by the decreased *IL2RA* (CD25) expression observed along with lower Tax expression (Inoue et al. 1986). Moreover, the results of our RNA decay assays using a METTL3/14 inhibitor found no changes in *IL2RA* mRNA stability. This suggests that observed decreases in *IL2RA* expression may be due to reduced Tax expression rather than the direct effect of the inhibitor. Because m^6^A participates in the global regulation of host as well as viral transcripts, inhibition of the METTL3/14 writer complex is expected to exert broader, transcriptome‐wide effects (Figure 3). Taken together, these findings indicate that m^6^A modifications contribute to the stability of *tax* and affect gene networks regulated by Tax.

In this study, we observed important differences in RNA stability between *tax* and *hbz*, both of which undergo m^6^A modification. As frequently reported in tRNA studies, RNA modifications can alter RNA secondary structures (Lorenz et al. 2017). For example, research on HIV‐1, a retrovirus that is similar to HTLV‐1, has shown that introducing mutations at m^6^A modification sites in RRE RNA, which prevent m^6^A modification, can alter its binding affinity to Rev, reduce its nuclear export efficiency, and decrease HIV‐1 replication levels (Lichinchi et al. 2016). Lichinchi et al. suggested that m^6^A modification might directly interact with Rev or otherwise change the secondary structure of RRE RNA, thereby affecting Rev binding. When taking these reports together, it is plausible that differences in m^6^A‐binding proteins, which depend on the presence and location of m^6^A modifications, can result in compromised RNA stability. According to Protein Atlas data (Uhlén et al. 2015), the gene expression levels of m^6^A reader proteins such as YTHDC1, YTHDC2, HNRNPA2B1, HNRNPC, and IGF2BP3 differ among activated CD4 T cells. However, although observed IGF2BP3 RNA levels were quite low, they were almost absent in Naïve CD4^+^ T cells but only slightly expressed in HTLV‐1‐infected HuT102 cells. Finally, since IGF2BP family proteins have been reported to contribute to the stabilization of m^6^A‐modified mRNA within the nucleus (Huang et al. 2018), IGF2BP3 might be involved in the stabilization mechanism of *tax* mRNA (Table S2). Since this study primarily relies on the METTL3/14 inhibition, the roles of m^6^A readers, including YTHDF1 and YTHDC1, may also be involved in regulating HTLV‐1.

In the future, identifying m^6^A modification sites within the HTLV‐1 genome, predicting secondary structures, and identifying major binding m^6^A readers will all be crucial for elucidating the comprehensive post‐transcriptional regulatory mechanisms mediated by m^6^A modifications in HTLV‐1. Therefore, understanding the gene expression mechanisms of HTLV‐1 genes may contribute insight into the mechanisms of HTLV‐1 latent infection and the development of infection prevention modalities.

## 4 Experimental Procedures

### 4.1 Cell Culture

The HTLV‐1‐infected cell line C91/PL and T cell line Molt4 were cultured in RPMI1640 medium (Thermo Fisher Scientific Inc., MA, USA). Lenti‐X 293T cells (Clontech Laboratories, CA, USA) were cultured in DMEM (Nissui Pharmaceutical Co. Ltd., Tokyo, Japan). Both media were supplemented with 10% FBS (Thermo Fisher Scientific Inc., MA, USA) and 1% Penicillin/Streptomycin (Thermo Fisher Scientific Inc., MA, USA). All cells were maintained in 5% CO_2_ at 37°C.

### 4.2 Establishment of METTL3 Knockdown Cell Line

To produce shMETTL3‐expressing lentivirus, 4 μg shMETTL3 lentiviral vector, 2.5 μg pCAG‐HIV‐gagpol, and 2.5 μg pCMV‐VSV‐G‐RSV‐rev were mixed in 1 mL Opti‐MEM (Thermo Fisher Scientific, Waltham, MA, USA) and 18 μL polyethylenimine (PEI) was added. After incubation for 20 min at room temperature, the mixture was applied to pre‐cultured HEK293FT cells and incubated at 37°C with 5% CO₂ for 4 h. The medium was then replaced with fresh DMEM containing 10% FBS, and cells were cultured for an additional 48 h. Supernatants were collected, passed through a 0.45‐μm filter, and concentrated by centrifugation at 12,000 × g for 2 h at 4°C. C91/PL cells were infected by spinoculation at 2000 × g for 2 h at 35°C. Infected cells were selected with blasticidin S (Thermo Fisher Scientific) to establish METTL3‐knockdown lines. The shRNA‐expressing lentiviral vectors targeted the following sequences: control (shCtrl), 5′‐CCTAAGGTTAAGTCGCCCTCG‐3′, shMETTL3#1, 5′‐GCTGCACTTCAGACGAATTAT‐3′, and shMETTL3#2, 5′‐GCAAGTATGTTCACTATGAAA‐3′. pLV‐EGFP:T2A:Bsd‐U6 were obtained from VectorBuilder (Chicago, IL, USA).

### 4.3 STM2457 Treatment

The METTL3/14 inhibitor STM2457 was purchased from Selleck Chemicals (TX, USA). Cells were collected 72 h after the addition of the inhibitor and subjected to RT‐qPCR (Final Conc. 0, 10, 25, 50 μM), WB (Final Conc. 0, 1, 10, 50 μM), RIP (Final Conc. 25 μM), RNA decay assay (Final Conc. 25 μM), FCS (Final Conc. 25, 50 μM), and RNA‐seq (Final Conc. 50 μM).

### 4.4 RT‐qPCR

Reverse transcription was performed using 4 μL of ReverTra Ace qPCR RT Master Mix (TOYOBO CO. LTD., Osaka, Japan) with 1 μg of RNA in a thermal cycler. For each well, 5 μL of THUNDERBIRD Next SYBR qPCR Mix (TOYOBO CO. LTD., Osaka, Japan), 0.25 μL of forward and reverse primers, 2.5 μL of dH_2_O, and 2 μL of the reverse transcription product were mixed. Gene expression levels were measured using a real‐time PCR system (Thermal cycler Dice, Takara Bio Inc., Shiga, Japan; CFX Duet Real‐Time PCR System, Bio‐Rad Laboratories Inc., CA, USA).

### 4.5 m^6^A RNA Immunoprecipitation (RIP)

Pre‐cultured cells were collected and extracted RNA using TRIzol reagent (Thermo Fisher Scientific Inc., MA, USA). Dynabeads Protein G (Thermo Fisher Scientific Inc., MA, USA) (50 μL) were washed with the RIP Buffer [10 mM Tris–HCl (pH 7.2), 150 mM NaCl, 0.1% NP‐40], then incubated with 2 μg of antibody control IgG (#2729), which was purchased from Cell Signaling Technology Inc. (MA, USA); Anti‐m^6^A antibody (#202003) was purchased from Synaptic Systems (Göttingen, Germany) for 10 min with gentle mixing to bind the Dynabeads and antibody. The cell lysate supernatant was added to the antibody‐bound magnetic beads and incubated with gentle mixing (4°C, Overnight) for immunoprecipitation. The beads were then washed four times with RIP Buffer, and the RNA bound to the magnetic beads was extracted and purified. RT‐qPCR was then performed to examine the presence of m^6^A modifications.

### 4.6 RNA Decay Assay

C91/PL cells exposed to the METTL3/14 inhibitor (STM2457) for 72 h were collected and seeded into a 12‐well plate at a density of 1 × 10^5^ cells/well. Actinomycin D (Nacalai Tesque, Kyoto, Japan) was added to achieve a final concentration of 5 μg/mL. Cells were then collected at 0 h (without Actinomycin D) and at 7, 24, and 48 h after the addition of Actinomycin D. RNA was extracted and subjected to RT‐qPCR.

### 4.7 Western Blotting

The cultured cells were washed with PBS and suspended in RIPA Buffer [10 mM Tris–HCl (pH 7.4), 1% NP‐40, 0.1% Sodium Deoxycholate, 0.1% Sodium Dodecyl Sulfate (SDS), 150 mM NaCl, 1 mM EDTA] supplemented with a protease inhibitor cocktail (Nacalai Tesque, Kyoto, Japan). The suspension was incubated on ice for 20 min, then centrifuged at 14,000 rpm for 20 min at 4°C, and the supernatant was collected. Protein concentration was measured using a Protein Assay (Bio‐Rad Laboratories Inc., CA, USA), and the protein supernatant was added to Sample Buffer [10% Glycerol, 3% SDS, 65 mM Tris–HCl (pH 6.8), 0.01% BPB (MeOH), 15% 2‐Mercaptoethanol] to achieve the desired concentration. After heating at 100°C for 5 min, the samples were spun down and electrophoresed on 12.5% acrylamide gels at 150 V for 1.5 h. The proteins were then transferred to a 0.2 μm PVDF membrane at 45 V for 2 h. The membrane was blocked with 5% skim milk/PBST [20 mM Tris–HCl (pH 7.5), 150 mM NaCl, 0.1% (w/v) Tween20] for 30 min at room temperature, and incubated with primary antibodies (Tax Ab clone: Lt‐4; HSP90 Ab #4874, Cell Signaling Technology Inc., MA, USA) overnight at 4°C. After washing three times with PBST, secondary antibodies (ECL Peroxidase labeled anti‐rabbit (#NA934VS); ECL Anti‐Mouse IgG, Horseradish Peroxidase linked whole antibody (#NA931V), Cytiva, Tokyo, Japan) were incubated for 1 h at room temperature. Following three washes with PBST, protein detection was performed using ECL Select Western Blotting Detection Reagent (Cytiva, Tokyo, Japan). Protein detection was carried out using Chemidoc (Bio‐Rad Laboratories Inc., CA, USA), and semi‐quantitative analysis was performed using ImageLab (Bio‐Rad Laboratories Inc., CA, USA) with the quantified data.

### 4.8 p19 gag ELISA

C91/PL cells were seeded at 1 × 10^4^ cells/well in a 24‐well plate and exposed to the METTL3/14 inhibitor (STM2457) for 72 h. After centrifugation (600 × *g*, 3 min), the supernatant was removed. The cells were then reseeded into a 12‐well plate and cultured with STM2457 for an additional 48 h. Following culture, the cells were centrifuged twice (1000 × *g*, 5 min), and the supernatant was collected for ELISA samples. ELISA was performed using the RETROTEK HTLV p19 Antigen ELISA (ZeptoMetrix, NY, USA) according to the manufacturer's protocol. After washing each well, samples diluted 500‐fold were added and incubated (37°C, 2 h). The wells were then washed six times with plate wash buffer, and the detector antibody was added and incubated (37°C, 1 h). After washing the wells, peroxidase working solution was added and incubated (37°C, 1 h), followed by another six washes. Finally, substrate solution was added and incubated (RT, 30 min), then stop solution was added, and absorbance at 450 nm was measured using the GloMax Explorer Multimode Microplate Reader (Promega, WI, USA).

### 4.9 CD25 Expression Analysis

After exposing C91/PL cells to the METTL3/14 inhibitor (STM2457) for 72 h, the cells were collected and centrifuged (1000 × *g*, 2 min), then washed with FACS Buffer. The cells were centrifuged again (1000 × *g*, 2 min), and the supernatant was removed. FACS Buffer (100 μL) was added, and the cells were stained with CD25‐APC (clone bc96, BioLegend, CA, USA) for 30 min at room temperature. After staining, the cells were centrifuged (1000 × *g*, 2 min), and the supernatant was removed. The cells were washed with FACS Buffer, and 7‐AAD (BioLegend, CA, USA) was added. CD25 expression was measured using the BD FACSymphony A1 (Becton, Dickinson and Company, NJ, USA). Measurement data were analyzed using FlowJo (v10.10) to calculate the Mean Fluorescence Intensity (MFI).

### 4.10 RNA‐Seq

Total RNA of C91/PL cells was extracted using TRIzol Reagent (Thermo Fisher Scientific Inc., MA, USA) and quantified and qualified by Agilent 2100 Bioanalyzer (Agilent Technologies, CA, USA), NanoDrop (Thermo Fisher Scientific Inc., MA, USA), and 1% agarose gel. Twenty nanograms of total RNA with RIN value above seven was used following library preparation. The library preparation and sequencing were processed and analyzed by GENEWIZ. The libraries with different indices were multiplexed and loaded on an Illumina HiSeq instrument according to the manufacturer's instructions (Illumina Inc., CA, USA). Sequencing was carried out using a 2 × 150 bp paired‐end (PE) configuration; image analysis and base calling were conducted by the HiSeq Control Software (HCS v2.2.38 or later) + OLB + GAPipeline‐1.6 (Illumina Inc., CA, USA) on the HiSeq instrument. For quality control, to remove technical sequences, including adapters, PCR primers, or fragments thereof, and quality of bases lower than 20, pass filter data of fastq format were processed by Trimmomatic (v0.30; Bolger et al. 2014) to be high‐quality clean data. For mapping, HISAT2 (v2.0.1; Kim et al. 2019) was used to index the reference genome sequence. Finally, clean data were aligned to the reference genome via software HISAT2. For differentially expressed gene analysis, HTSeq (v0.6.1; Anders et al. 2015) estimated gene and converted read counts to transcripts per million (TPM) from the pair‐end clean data. Gene Ontology analysis was performed by DAVID Bioinformatics Resources (https://david.ncifcrf.gov/; Huang et al. 2009; Sherman et al. 2022).

### 4.11 MeRIP‐Seq Secondary Analysis

Sequence data were retrieved from the DDBJ Sequence Read Archive (SRA) in SRA format (accession numbers: SRR2648293, SRR2648294, SRR2648296, SRR2648297) and converted to FASTQ files using SRA‐tools (v2.9.6; https://github.com/ncbi/sra‐tools). Quality control and adapter trimming were performed using fastp (v0.23.2; Chen et al. 2018). The processed reads were mapped to the human reference genome (hg38, obtained from NCBI) using HISAT2 (v2.2.1; Kim et al. 2019). Peak calling was conducted using MACS2 (v2.2.7.1; Zhang et al. 2008).

### 4.12 Data Analysis

Statistical significance was determined using Student's *t*‐test. All statistical analyses were performed using the statistical software R (ver 4.4.1; R Core Team 2024) or Microsoft Excel. Time series data from the RNA decay assay were analyzed using generalized linear modeling (GLM) with a Gamma distribution to accommodate non‐normality and heteroscedasticity. Statistical significance was assessed by calculating 95% confidence intervals. Integrative Genomics Viewer (IGV) tool was used for visualizing and interpreting the results of ChIP‐seq.

## Conflicts of Interest

The authors declare no conflicts of interest.

## Supporting information


**Figure S1:** Results of KEGG PATHWAY analysis. Genes compatible with the “human T‐cell leukemia virus 1 infection” pathway are highlighted with asterisks.

**Table S1:** List of differentially expressed genes under STM2457 treatment in C91/PL cells.
**Table S2:** Normalized TPM data of m6A‐related genes in activated naive CD4+ T‐cells and HTLV‐1‐infected cell line HuT102.

## Data Availability

The data that support the findings of this study are available from the corresponding author upon reasonable request.
