# Supplementary material for: m6A RNA Modification Controls HTLV‐1 Tax and Host Gene Expression
Source: Genes Cells. 2025 Oct 7;30(6):e70054. doi: 10.1111/gtc.70054 (PMC12504147; doi:10.1111/gtc.70054)
Supplement: Supplementary file 1 — Figure S1: Results of KEGG PATHWAY analysis. Genes compatible with the “human T‐cell leukemia virus 1 infection” pathway are highlighted with asterisks. [file GTC-30-0-s001.pdf]

Supplementary Figure 1

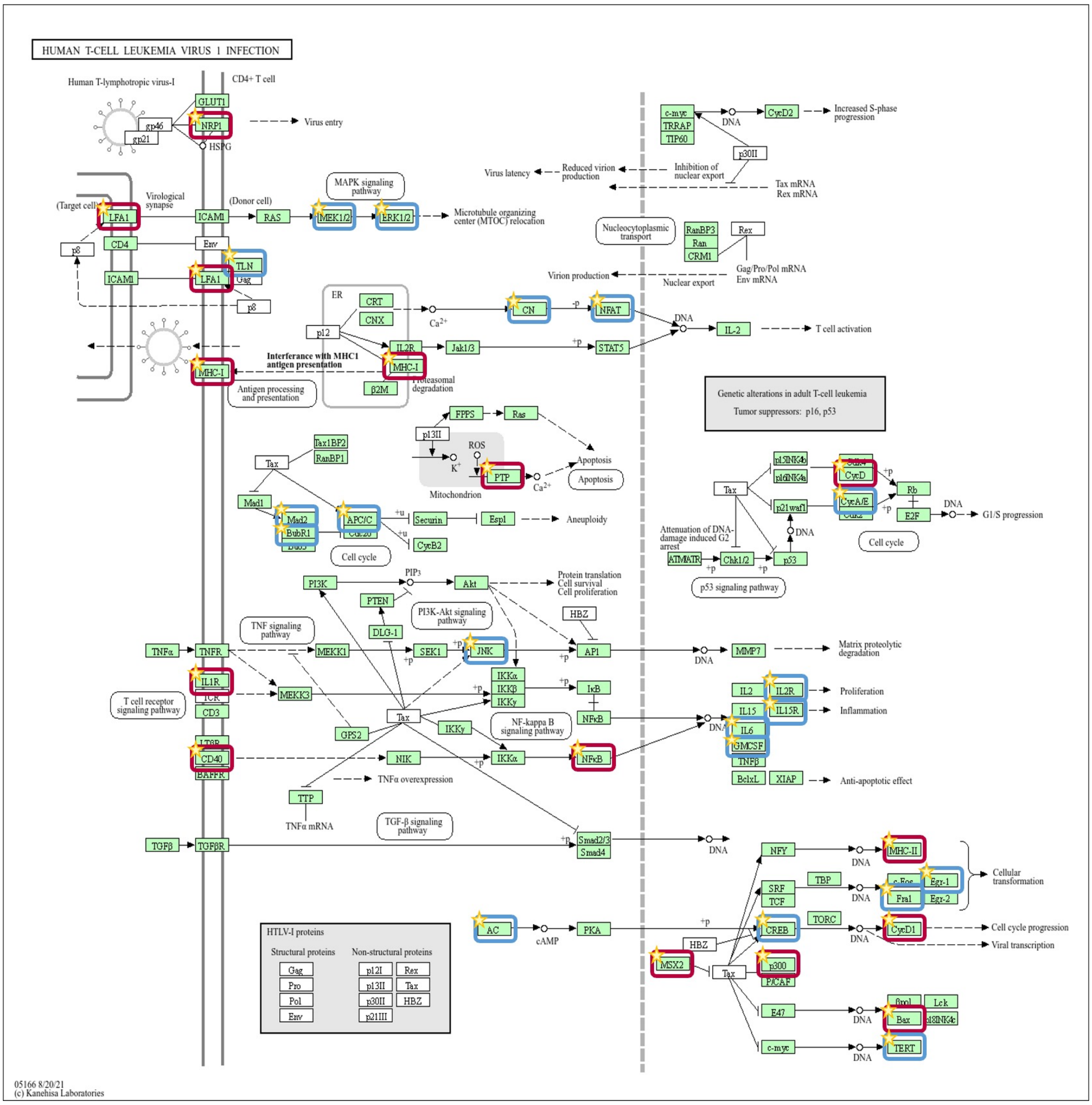

Results of KEGG PATHWAY analysis. Genes compatible with the 'human T-cell leukaemia virus 1 infection' pathway are highlighted with asterisks.
